# Dr. Selden H. Talcott

**Published:** 1902-07

**Authors:** 


Dr. Selden H. Talcott, Superintendent of the State Homeopathic Hospital, at Middletown, died June 15, 1902, aged 60 years. Dr. Talcott had been the head of the institution for 25 years and was widely known as an authority on nervous diseases and as an alienist. He had been ill but three weeks.

